# Methodological issues regarding power of classical test theory (CTT) and item response theory (IRT)-based approaches for the comparison of patient-reported outcomes in two groups of patients - a simulation study

**DOI:** 10.1186/1471-2288-10-24

**Published:** 2010-03-25

**Authors:** Véronique Sébille, Jean-Benoit Hardouin, Tanguy Le Néel, Gildas Kubis, François Boyer, Francis Guillemin, Bruno Falissard

**Affiliations:** 1 EA 4275 "Biostatistique, recherche clinique et mesures subjectives en santé", Faculté de Pharmacie, Université de Nantes, 1 rue Gaston Veil, 44035 Nantes Cedex 1, France; 2 Plateforme de Biométrie, Cellule de promotion de la recherche clinique, CHU de Nantes, France; 3 University of Reims Champagne-Ardenne, Faculty of medicine - EA 3797, 35 rue Cognacq Jay, F-51095, Reims, France; 4 Department of Physical Medicine and Rehabilitation, Sebastopol Hospital, 48 rue de Sébastopol, F-51092, Reims, France; 5 Nancy-Université, Paul Verlaine Metz University, Paris Descartes University, EA 4360 Apemac, Nancy, France; 6 INSERM 669, Université Paris-Sud, and Université Paris Descartes, Paris, France; 7 AP-HP, Hôpital Paul Brousse, Département de santé publique, Villejuif, France

## Abstract

**Background:**

Patients-Reported Outcomes (PRO) are increasingly used in clinical and epidemiological research. Two main types of analytical strategies can be found for these data: classical test theory (CTT) based on the observed scores and models coming from Item Response Theory (IRT). However, whether IRT or CTT would be the most appropriate method to analyse PRO data remains unknown. The statistical properties of CTT and IRT, regarding power and corresponding effect sizes, were compared.

**Methods:**

Two-group cross-sectional studies were simulated for the comparison of PRO data using IRT or CTT-based analysis. For IRT, different scenarios were investigated according to whether items or person parameters were assumed to be known, to a certain extent for item parameters, from good to poor precision, or unknown and therefore had to be estimated. The powers obtained with IRT or CTT were compared and parameters having the strongest impact on them were identified.

**Results:**

When person parameters were assumed to be unknown and items parameters to be either known or not, the power achieved using IRT or CTT were similar and always lower than the expected power using the well-known sample size formula for normally distributed endpoints. The number of items had a substantial impact on power for both methods.

**Conclusion:**

Without any missing data, IRT and CTT seem to provide comparable power. The classical sample size formula for CTT seems to be adequate under some conditions but is not appropriate for IRT. In IRT, it seems important to take account of the number of items to obtain an accurate formula.

## Background

Many clinical studies attempt to incorporate measure of important characteristics, such as health related Quality of Life (QoL), anxiety, depressive symptoms, fatigue, addictive behaviours using Patient reported outcomes (PRO), in order to measure endpoints that reflect patient's perception of his or her well-being and satisfaction with therapy. QoL and other perceived health measures (pain, fatigue, ...) are increasingly used as important outcomes in clinical trials and medical surveillance and are considered as highly valued endpoints of medical care [1].

PRO differ from other measurements because such patient's characteristics cannot be directly observed and measured and are usually evaluated using self-assessment questionnaires which consist of a set of questions often called items whose responses provided by the patients are frequently combined to give scores. Two main types of analytical strategies can be used for such data: so-called classical test theory (CTT) and models coming from Item Response Theory (IRT). CTT relies on the observed scores (possibly weighted sum of patients items' responses) that are assumed to provide a good representation of a "true" score, while IRT relies on an underlying response model relating the items responses to a latent parameter, often called latent trait, interpreted as the true individual QoL, for instance. Such IRT models also take into account some items parameters.

Methods coming from modern measurement theory, such as IRT models, might provide a powerful framework to build and reduce PRO instruments and analyse such data in an efficient and reliable manner and should provide valid measures of QoL, anxiety, or pain for instance [2]. IRT can improve on the classical approach to PRO assessment with advantages that might sometimes include appropriate management of possible floor and ceiling effects, comparison of patients across different instruments, interval measurements on the latent trait scale. Indeed, models coming from IRT are more and more used for the construction, validation and reduction of questionnaires [3,4], in particular in the framework of the Patient-Reported Outcomes Measurement Information System (PROMIS) network [5] for creating item banks. Consequently, many PRO instruments are found to be well adapted to IRT modelling either because of the way they were developed using such IRT-based strategies or because of their desired psychometric properties.

In most of current literature of intervention and observational studies, the choice of a statistical strategy for PRO data analysis is more often based on CTT and occasionally on IRT and seems to be driven to date by the researchers' practice and familiarity with one approach or another. However, if IRT models best describe data coming from some PRO instruments, such as QoL questionnaires, one may wonder whether they should provide a better and more powerful strategy than CTT to detect clinically meaningful effects. In the case of the comparison of QoL levels between two independent groups of patients, power will depend in particular on the difference in the means of QoL levels and the standard deviation of one of the groups (or pooled standard deviation), often combined together into the concept of effect size (difference in means over the standard deviation). The required sample size to detect a pre-specified effect size with type I error (α) and power (1-β) is often computed during the planning of studies. The larger the effect size, the larger the power for a given sample size. In all cases, an appropriate analytical strategy and an accurate estimation of the effect size from data is required to achieve the desired power. Hence, if an IRT model fits the data coming from some QoL questionnaire well, the precision of the estimated effect size using this model might be higher than the one provided using CTT analysis. However, whether one of these approaches is more powerful than the other remains unknown especially for IRT that often incorporates many parameters that might either be fixed as known constants or estimated. These issues all have strong implications for sample size planning as well as for the analysis of PRO data and were investigated through a simulation study. The purpose of our work was therefore to study the statistical properties of CTT and IRT by simulations regarding power and corresponding effect sizes, to compare these methods between them and to provide some guidelines for sample size determination for studies evaluating PRO.

## Methods

### IRT modelling

Some of the commonly used IRT models are the Rasch model for binary responses [6,7], and the Rating Scale model or the Partial Credit model for multiple (> 2) response options [8,9]. We shall mainly consider the Rasch model, extensions of the results to other IRT models will also be discussed.

In IRT models and in the Rasch model in particular, the responses to the items are modelled as a function of a latent variable representing the so-called ability of a patient measured by the questionnaire (e.g. QoL, anxiety, fatigue, ...). Usually, the sample of patients is believed to be representative of a more general population, and the latent variable is then considered as a random variable often assumed to follow a normal distribution. In this model, each item is characterized by one parameter (δ_j _for the jth item), named difficulty parameter because the higher its values, the lower the probability of positive (favourable) responses of the patient to this item regarding the latent trait being measured.

Three assumptions govern these models: (i) Unidimensionality: one latent trait influences the responses to all the items, (ii) Local independence: for a given individual, the responses to the items are independent, and (iii) Monotonicity: the probability to have a positive response to a given item does not decrease with the latent variable.

Let us consider that N patients have answered a questionnaire containing J binary items and let X_ij _be the random variable representing the response of patient i to item j with realization x_ij_, and θ_i _be the realization of the latent trait θ for this patient.

For each patient, the probability of responding to each item is:

where δ_j _represents the difficulty of item j.

We consider the latent variable θ as a random variable following a normal distribution with unknown parameters μ and σ^2^. Using the local independence assumption, the marginal likelihood can be written down and the person parameters (parameters of the distribution of the latent trait) can be jointly estimated with the item parameters by marginal maximum likelihood estimation (MML) obtained from integrating out the random effects [6].

### Sample size determination in the framework of normally distributed endpoints

Suppose we plan to conduct a cross-sectional study aiming at comparing two independent groups on an endpoint assumed to be normally distributed with common variance σ^2^. Under these conditions, the well-known formula for the comparison of normally distributed endpoints in a two independent group study can be used.

Let the study objective involve the comparison of the two hypotheses: H_0_: μ_1 _= μ_2 _against H_1_: μ_1 _≠ μ_2_, where μ_1 _and μ_2 _represent the population means in the first and second group, respectively. The effect size (ES) can be computed as [10].

The conventional sample size formula for a two-sided test size at α and a desired power at 1 - β is the following, assuming n_1 _patients in one group and n_2 _in the other group:

which can be generalized to:  with Z_η _denoting the 100η th percentage point of the standard normal cumulative distribution. In practice, σ^2^, μ_1 _and μ_2 _are unknown population parameters and initial estimates based on the literature or pilot studies are often used for calculations. For instance, using this formula, about 100 patients (n_1 _= n_2 _= N = 105) are required per group to detect an effect size of 0.5 with 95% power and a 5% type I error in a two-sided test.

### Sample size determination in an IRT framework

Suppose we are willing to plan a similarly designed two-group cross-sectional study using an IRT model and that the outcome of interest is, for instance, one of the aspects of quality of life based on a given dimension of a questionnaire. Since the latent trait is assumed to follow a normal distribution, we shall make the assumption that the above-mentioned formula is also well suited for sample size calculation based on the latent trait distribution.

In the framework of IRT, let θ be the latent trait (focused aspect of quality of life) with normal distributions N(μ_IRT1_, σ^2^_IRT_) and N(μ_IRT2_, σ^2^_IRT_) in the first and second group, respectively.

Assuming that the study now involves the comparison of the two hypotheses: H_0_: μ_IRT1 _= μ_IRT2 _against H_1_: μ_IRT1 _≠ μ_IRT2_, the effect size on the latent trait scale can be computed as . If we use the same sample size formula at α and β levels, we get:

We may notice that, as before, σ^2^_IRT_, μ_IRT1 _and μ_IRT2 _are unknown population parameters with the additional particularity that they now characterise an unobserved latent variable.

### Simulation study

A thousand cross-sectional studies were simulated for the comparison of two groups at a point in time on a PRO measure containing binary items (0/1). The PRO measure was assumed to follow a Rasch model. The latent trait level for the patients in the first group θ_1 _was assumed to follow a normal distribution with mean μ_IRT1 _and variance σ^2^_IRT _and the latent trait in the second group θ_2 _was assumed to follow a normal distribution with mean μ_IRT2 _= μ_IRT1 _+ d and same variance. The study involved the comparison of the two previous hypotheses which can now be expressed in the following way: H_0_: d = 0 against H_1_: d ≠ 0, giving the corresponding effect size: . All datasets were simulated under H_1 _with different effect sizes on the latent trait (ES_IRT_).

In order to reflect the range of effect sizes, samples sizes, and number of items often encountered in clinical and epidemiological research in a variety of situations, data were simulated using the following values. Furthermore, to better capture the influence of the sample size and of the number of items on power, larger ranges than usually encountered in clinical research were investigated for both.

- ES_IRT_: 0.2 (small), 0.5 (medium) or 0.8 (high) according to Cohen [10]

- sample size per group: N = 100, 200, 300, 400, 500 or 800

- number of items: J = 5, 10, 15, 20, 50 or 100

- variances of the latent traits: σ^2^_IRT _= 1

- difficulty parameters: quantiles of a standardized normal distribution

- μ_IRT1 _is determined by using  (as a consequence, μ_IRT2 _= d/2)

In each simulated dataset, the responses to the items have been generated using a Rasch model, in which a group effect was incorporated. Since the variance of the latent trait is fixed to 1, the group effect parameter is equal to d. Let G_i _the random variable representing the group of the ith individual and g_i _its realization (coded as 0 or 1 for the first and second group, respectively) and let d be the group effect parameter:

For each simulated dataset, the parameters d and σ^2^_IRT _were estimated using marginal maximum likelihood. The μ_IRT1 _and δ_j _parameters could be considered either as known or as unknown parameters (and were estimated in this case). In the former case, for the δ_j_s, a precision parameter ε was used in the simulations in the following way:

where Unif represents the uniform distribution, δ_j _* denotes the known item parameter used in the Rasch model for data analysis and δ_j _is the parameter used for simulating the data.

Two possibilities were investigated:

▪ The parameters δ_j _are known with a good precision and ε = 0.0, hence δ_j _* = δ_j_

▪ The parameters δ_j _are known with a moderate or poor precision and ε varies from 0.1 to 1 (with values 0.1, 0.25, 0.5, 0.75, and 1.0)

The possibility to fix μ_IRT1 _and δ_j _might correspond to three practical kinds of situations:

▪ **Situation 1**: the μ_IRT1 _and δ_j _parameters are known and fixed. This might correspond to a randomized clinical trial where the questionnaire has been validated by an IRT model in exactly the same population than the studied population.

▪ **Situation 2**: only the δ_j _parameters are known and fixed (μ_IRT1 _is estimated). This might correspond to a study where the questionnaire has been validated by an IRT model in a population close to the studied population: the properties of the questionnaire are assumed to be similar in both populations; nevertheless, we suppose that the studied population can display a shift on the mean of the latent trait as compared to the population of validation, and this shift is estimated.

▪ **Situation 3**: none of the parameters are known and fixed (μ_IRT1 _and δ_j _parameters are estimated). This corresponds to a study where the questionnaire has already been validated using classical methods (face validity, reliability, structure validity...), but not using an IRT model.

The test statistic of group effect used for IRT was d and its statistical significance was assessed by a Wald test.

In order to compute the power of CTT and to compare CTT and IRT in terms of power and corresponding effect sizes, the score for each patient was calculated as the unweighted sum of his/her responses to the items. Since the data have been simulated using an IRT model, the effect size on the latent trait scale (ES_IRT_) was known whereas the effect size on the score scale (ES_CTT_) had to be calculated from the simulated data. It was computed using the estimated mean scores in each group ( and ) along with the estimation of the global standard deviation . The effect size for CTT was then estimated as . The comparison of the mean scores between groups was performed using a two-sample t test.

For IRT and CTT approaches, the powers were computed as the proportion of significant group effects among the 1,000 simulated datasets. The power and effect sizes obtained using IRT modelling (Rasch model) or CTT were compared and the major parameters having the strongest impact on them were identified. The datasets were simulated using the -simirt- module of the Stata 11 package [11] and analysed using the -raschtest- module [12].

## Results

### Simulation study

#### Situation 1 (δ_j _and μ_IRT1 _are known and fixed)

The power achieved by the tests of group effects using IRT modelling (Rasch model) with fixed μ_IRT1 _and δ_j _parameters (j = 1, ..., J) with different levels of precision for the latter as compared with their simulated values are given in additional file 1 for different values of the effect sizes on the latent trait ES_IRT_, sample sizes per group N, and number of items J.

The power was of the same order of magnitude (for J = 5) or even higher (for J > 5) than the expected power using the well-known formula for normally distributed endpoints based on the corresponding ES_IRT _and sample size per group N. The impact of the precision of the fixed item difficulty parameters on power was moderate but noticeable, especially for small N and J, the power decreasing as ε increased. Indeed, the maximum decrease in power was observed for ES_IRT _= 0.2, N = 100 and J = 5: as compared with an expected power of 0.293, a 6%, 11%, and 16% decrease in power was noticed with a good, moderate or poor precision of the item difficulty parameters, respectively. Moreover, this decrease was significant; the 95% confidence interval of the power mean did not include the expected value of 0.293 when ε ≠ 0. A strong impact of the number of items J was also observed on power. It increased with J and was larger than the expected power as soon as J was at least equal to 5 items, whatever the precision, the increase in power being more marked when ES_IRT _= 0.2 and for small N. Indeed, as compared with the corresponding expected power of 0.293, for an ES_IRT _= 0.2 and N = 100, a 27% and 72% increase on average in power was observed when J = 10 and 100 items, respectively. The power was close to its maximum value of 1.000, so only a corresponding 2% increase in power was observed when N = 800 when J ≥ 10 items. The power reached its maximum value of 1.000 as soon as ES_IRT _was higher than 0.5 and N larger than 200.

#### Situation 2 (δ_j _known and fixed, μ_IRT1 _is estimated) and situation 3 (δ_j _and μ_IRT1 _are estimated)

**Figure 1:**
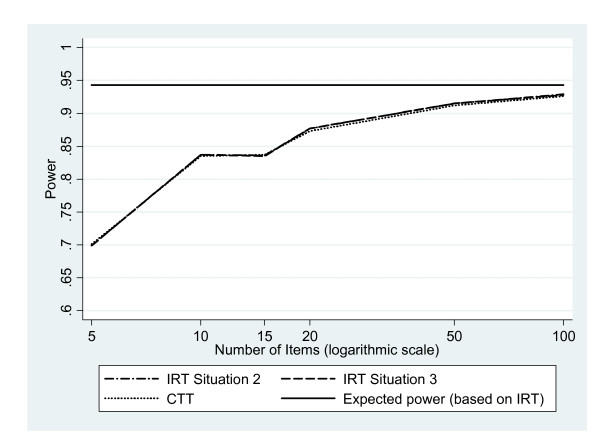
**Power achieved by the tests of group effects using IRT or CTT**. Description: Evolution of the power achieved by the tests of group effects using IRT (Rasch model) or CTT (classical test theory) as a function of the number of items of the questionnaire for an effect size on the latent trait ES_IRT _= 0.5 and N = 100 patients per group. IRT: item response theory. Situation 2: unknown person parameters and known item parameters; Situation 3: unknown person and item parameters.

The power achieved by the tests of group effects using IRT modelling (Rasch model) or CTT approaches are given in figure 1 for both situations for an ES_IRT _= 0.5 on the latent trait and N = 100 patients per group, and in additional file 2 for all investigated effect sizes ES_IRT _for different values of sample sizes per group N and number of items J.

When μ_IRT1 _was estimated, the power was of the same order of magnitude using either IRT modelling or CTT for all values of N and J, whether δ_j _were assumed to be known and fixed or not. Furthermore, the precision associated with the item difficulty parameters did not have an impact on power (data not shown). Moreover, the power using either method was most of the times much lower than the expected one based on the effect size on the latent trait, ES_IRT_, except for large ES_IRT_, N and J. For instance, as compared with an expected power of 0.942, for N = 100 patients per group and ES_IRT _= 0.5, an approximate 26% decrease in power was observed for IRT and CTT when J = 5. This decrease reduced to 2% when J = 100. About N = 200 patients per group was required to reach a power of at least 0.900 when ES_IRT _= 0.5. As expected, the impact of the effect size ES_IRT _and sample size per group N was observed with a rise in power as they increased. A strong impact of the number of items J was also observed, the increase in power being more marked for small ES_IRT _and N. Indeed, for an ES_IRT _= 0.2 when N = 100, an 86% increase in power (from 0.145 to 0.270) was observed as the number of items rose from J = 5 to J = 100. A corresponding 23% increase in power (from 0.790 to 0.970) was observed when N = 800. For an ES_IRT _= 0.5, the increase in power was either 33% or 1% for N = 100 or 300, respectively, as J increased from 5 to 100 items. Almost no increase was observed for N ≥ 400. For an ES_IRT _= 0.8, the power rapidly reached its maximum value of 1.000, only a slight increase in power (2%) was observed for N = 100 when J rose from 5 to 50 items.

The mean effect size for CTT, computed from the simulated data (1,000 simulations) as previously described, is given in additional file 3 for different values of the ES_IRT _on the latent trait, sample sizes per group N and number of items J. The mean effect size on the score scale was always lower on average than the corresponding effect size on the latent trait scale for all values of the number of items J or sample size per group N. It increased with the number of items J for all N. Indeed, for all values of the ES_IRT _(0.2 to 0.8), when J = 5 items or J = 100 items, the mean effect size on the score scale represented on average 69% or 94% of the corresponding effect size on the latent trait scale, respectively. Moreover, in most of the cases, the 95% confidence intervals of the mean of the effect size of the score (data not shown) did not include the corresponding expected value on the latent trait scale, except for ES_IRT _= 0.2, N < 200 and J = 100.

### A practical example using NHP data

We illustrate the results of the simulation study with an example coming from a pilot study whose data were used for sample size calculations for the planning of a future larger study. The main objective of the upcoming study is to compare the level of pain between two groups of patients having muscular dystrophies. The first group concerns patients with Steinert disease, and the second group concern others muscular dystrophies, mainly Duchenne's and Becker's muscular dystrophies. The ethics committee of Reims, France granted approval for the study.

In the preliminary study, patients were recruited from the university hospital of Reims, 52 patients were included with Steinert's disease and 95 patients with others muscular dystrophies. The Nottingam Health Profil (NHP) questionnaire was used in order to evaluate the global Quality of Life of the patients and the main outcome was the score on the pain subscale. The latter is composed of 8 binary items and the score is calculated as a weighted sum of the items, according to the NHP manual.

Patients completed the NHP at inclusion in the study. The mean scores for the pain subscale in each group were 32.9 (σ^2 ^= 27.9^2^) for patients with a Steinert's disease and 25.5 (σ^2 ^= 26.6^2^) for patients with other diseases (the global variance = 27.3^2^). Consequently, the effect size on the weighted score was: (32.9-25.5)/27.3 = 0.271. The test of the difference between the mean scores using a Student's test was not significant (p = 0.15).

A mixed Rasch model including a group effect was fitted on these data. The global fit of the Rasch model was not rejected by the R1m test (p = 0.329) [13]. The estimations of the difference between the mean levels of the latent trait of the two groups of patients was 0.649 and the variance of the latent trait was 1.983^2 ^(non significant difference between groups: p = 0.08). Consequently, an estimation of the effect size on the latent trait was: 0.649/1.983 = 0.327. When computing the required sample size using these data and the classical formula with a type I error of 5% and a power of 90%, 287 patients per group are required using the effect size of the score scale, and 195 patients per group using the effect size obtained with the Rasch model, on the latent trait scale.

In order to evaluate the relevance of these sample sizes, a Rasch model was used to simulate datasets with 287 or 195 patients per group. The Rasch model parameters were randomly drawn in the 95% confidence interval previously estimated on the pilot study data. This strategy somehow stands between situations 2 and 3 where items parameters are estimated taking into account previous knowledge provided by the data. A thousand datasets were generated using these values for each calculated sample size. For IRT and CTT approaches, the powers were computed as the proportion of significant group effects among the 1,000 simulated datasets, using a Rasch model including a group effect for the former and a t-test on the weighted scores between the two groups, for the latter. The estimated powers are given in additional file 4 for 195 and 287 patients per group.

In accordance with the simulation study, we observed that the estimated effect size was smaller on the score scale than the one estimated using the Rasch model, on the latent trait scale (0.271 vs 0.327). Moreover, the required sample size computed using the effect size on the latent trait did not allow obtaining the expected power (78.9% instead of 90%), stressing the fact that the usual formula might not be well-suited for IRT models. In addition, the required sample size computed using the effect size based on the score allowed obtaining the expected power, whatever the method of analyse of the data (score or Rasch model). Consequently, for this study, it seemed to be reasonable to propose to include 287 patients per group in a future trial aiming to evaluate the difference concerning the perception of pain between patients with a Steinert's disease and the patients with another muscular dystrophy.

## Discussion

We investigated and compared the power and corresponding effect sizes of two main approaches for the analysis of PRO data, namely CTT and IRT-based methods. For the latter, different scenarios were investigated according to whether items and/or person (mean of the latent trait) parameters were assumed to be known (situations 1 and 2), to a certain extent for item parameters, from good (as in some randomized clinical trials for instance) to poor precision, or unknown and therefore had to be estimated (situation 3).

When items and person parameters were both assumed to be known in the Rasch model (situation 1), the power was either of the same order of magnitude or even higher than the expected one using the regular sample size formula as the number of items increased. The impact of the precision of item parameters values was modest and mainly perceptible for high values of ε (ε = 1.0), corresponding to a rather poor precision that might be rarely encountered in such a context. Indeed, using known and prespecified person and item parameters can be envisaged in randomized clinical trials aiming at evaluating PRO where IRT-validated instruments are used. This situation corresponds to the most favourable one regarding power; however it has to be stressed that assuming both person and items parameters to be known implies that the patient population in the trial is similar to the one used for validating the instrument, which can be restrictive. Indeed, the fact that there isn't any shift on the mean of the latent trait between the two populations often remains quite uncertain.

When person parameters were assumed to be unknown and items parameters to be either known (situation 2) or not (situation 3), the power achieved by the tests of group effects using IRT modelling (Rasch model) or CTT were similar in all situations and always lower than the expected power using the well-known sample size formula for normally distributed endpoints based on ES_IRT _(except for large ES_IRT_, N and J). Moreover, the number of items J had a substantial impact on power for both methods, the power increasing quite importantly with J. In light of the observed results, smaller power than anticipated for both methods can possibly be explained as coming from two different phenomena according to the chosen approach. For CTT, we also observed that the effect sizes on the score and on the latent trait scales were different and always lower for the score based on CTT. We can recall that sample size calculation was only based on ES_IRT _and not on ES_CTT _(which was calculated from the simulated data). Since ES_CTT _was in fact lower than ES_IRT_, the sample size required for CTT to detect a smaller effect size than expected had to be larger than the one calculated for IRT based on ES_IRT_. Hence, the power for CTT was consequently lower than the expected one for IRT.

For IRT, the loss in power might be related mostly to the precision of the latent trait mean estimation. In fact, whether item parameters were assumed to be known exactly (good precision, ε = 0.0) or not had no impact on power, which was similar in all situations, including CTT. In all cases, marginal maximum likelihood estimation provided, as expected, unbiased estimates of the mean of the latent trait (μ_IRT1_) and of item parameters, when needed. However, the impact on power of the uncertainty of the estimation of μ_IRT1_, reflecting inter-individual variability, underlines the importance of taking it into account in the model. The number of items also had a strong impact on power that was observed throughout all of our results whatever the method used (CTT or IRT with known or unknown item parameters). In CTT, this number seems to be indirectly taken into account when calculating the effect size (the value of the effect size getting bigger as the number of items increases). Consequently, the expected power in CTT is correctly computed with the traditional formula using the effect size on the score scale. This result was expected because the score is an observed variable, even if the traditional formula requires some specific conditions to be valid as normality of the score which was actually artificially created in our study by letting the difficulty parameters of the items to be regularly spaced.

In IRT, the important effect of the number of items has strong implications, notably for study design and sample size calculation. Indeed, it seems that the number of subjects required for detecting a given effect size on the latent trait scale with some specified power can be greatly modified according to the number of items considered. For instance, for an ES_IRT _of 0.2, if one wishes to achieve a power of about 80% using IRT with unknown person parameters (situations 2 and 3), 800 patients are needed in each group if the number of items J = 5, an equivalent power size can be obtained with only 500 patients per group if J = 20 items. Similar results of the J effect are obtained when using the Rasch model with fixed person and items parameters (situation 1) with much fewer patients required to detect the same ES_IRT _with equivalent power: about 400 patients are required for J = 5 items and only about 300 patients if J ranges between 10 and 15 items. Such an effect of J on power and on sample size requirements should clearly be taken into account in the sample size formula for PRO which is not the case at this time. The latter should indeed incorporate some parameters related to the number of items of the dimension one is willing to study.

Some guidelines for sample size determination for two-group cross-sectional comparisons of PRO have been suggested in the framework of CTT [14] and a simulation study has provided some insights for sample size planning using IRT [15]. In the CTT framework, several sample size formulas have been proposed assuming either normally distributed continuous data, continuous data using non-parametric methods, ordinal data [16], or bootstrap sample size estimation. Even though all of these methods do not take into account the number of items used to assess the patients explicitly, we can hypothesise that it is indirectly taken into account through the influence on the difference between the mean scores of the two groups and the value of the variance of the score (with an increase for both as the number of items does). Since the score is observed and does not necessitate a model for its estimation, it can be considered as a usual variable which can be directly measured (biological markers, temperature, blood pressure....) and the formula used to determine the sample size can still be the usual formula under some conditions [14] such as normality, proportional odds, ....

In the IRT framework, the difference between the means of the latent trait in the two groups as well as the variance is not influenced by the number of items (even if the precision of these estimations are better with a larger number of items). Nevertheless the power seems to be improved with large questionnaire. Consequently, the usual formula to determine the sample size seems to be inadequate, because it does not take into account the number of items, but only the means and the variance of the latent trait (whose values do not depend on the number of items). This inadequacy can be explained by the latent nature of the latent trait; it is not an observed variable and its estimation requires the use of a model which creates uncertainty on a large number of parameters (parameters of the distribution of the latent trait and possibly items parameters).

The work of Holman et al. [15] extended recently by Glas et al. [17] have already stressed the impact of the size of the studied questionnaire on power and hence, sample size requirements, but the expected power based on the mean difference of the latent traits was not computed. Furthermore, they have also recalled that large sample sizes (> 1000) are usually required to estimate the items parameters of an IRT model with adequate precision which is much more often encountered in educational surveys than in clinical or epidemiological research. However, they have noted as we did in our study that small and moderate effect sizes can be detected with reasonable power even with small sample sizes provided that the uncertainty of the latent variable is taken into account. Holding items parameters fixed does not enhance power, except when person parameters are also assumed to be known, which might be too restrictive as already discussed. However, fixing items parameters to known values coming from previous validation studies that integrated IRT models might be interesting because it allows the comparison of patients coming from different studies that made use of the same instrument. Furthermore, such items parameters do not constitute the main interest in clinical studies which are more concerned with latent trait level estimation and corresponding effect sizes. Hence, this raises the question of the way of handling these parameters, should they be considered as nuisance parameters, should they be fixed at some plausible values coming from published items banks in the literature? More studies are needed to answer these important questions that have strong implications for sample size planning and statistical analysis of PRO.

Several limitations of our study and further necessary developments can be underlined. Our work focused on one of the most well-known IRT model, the Rasch model, other models well suited for the analysis of polytomous item responses [6] such as the Partial Credit Model or the Rating Scale Model should also be studied. Indeed, the number of modalities per item as well as the number of items could also have an important impact on power. Another field of investigation that has not been covered by our study is the impact of a non symmetrical score. Indeed, in the present study, data were simulated by adjusting the difficulty of the items on the mean value of the latent trait (the mean difficulty parameters was equal to the mean of the simulated latent trait). More, the items parameters were fixed regularly following the standardized normal distribution. Consequently, the obtained score had a distribution close to a normal, which constitutes an optimal condition to use the CTT approach that not always reflects practical situations. Simulation of data leading to a non-normal score could be performed and allow determining the robustness of the CTT approach to the violation of the normality assumption.

Moreover, in a statistical analysis perspective, the impact on power of missing values possibly non ignorable is also an interesting and necessary extension, because such incomplete data are often encountered in PRO studies. Since the Rasch model allows estimating the parameters by using all the available information based on the likelihood, we might expect a better performance of IRT as compared to CTT in this framework. Indeed, the score cannot be computed for individuals with one or more missing value, unless using missing value imputation. The impact of the number of missing values, their potential informativity and of the imputation method is an important topic for future research.

Furthermore, other study designs and in particular longitudinal data settings should be part of further investigations in this domain since PRO data are often gathered in this way in order to investigate time, group effect as well as possible interactions between them or other covariates. Last, in the framework of PRO data, adaptive and sequential designs eventually incorporating samples size re-estimation [18] might also offer a valuable tool by using accumulating data to decide how to modify some aspects of a study as it continues without undermining its validity and integrity. Whether IRT or CTT-based approaches would offer the best alternative in this context is not known at this time.

## Conclusions

The following advice can be proposed for sample size and analysis issues for PRO data: i) one should avoid using prespecified and fixed person parameters since patients included in the study of interest and in the validation study are very likely to differ from one another, even slightly, and this may lead to a substantial loss in power, ii) using prespecified and fixed items parameters coming from IRT-based validated instrument might be valuable, even if it does not improve power, because it takes benefit from one of IRT's strength, that is letting the possibility to compare patients from different studies that have used an instrument with similar psychometric properties, iii) one can, under some conditions, use the classical sample size formula for CTT (since the score can be considered as an observed variable) and validate this sample size estimation with IRT using simulations as was done for the NHP data.

However, applying this formula directly in an IRT framework is not appropriate since the latent variable is an unobserved variable whose estimation requires a model which creates uncertainty. Moreover, in the IRT approach, more research is needed and preliminary work (Hardouin J-B, Amri S, Sébille V. Towards sample size calculations for item response theory analysis for the comparison of two groups of patients, submitted) also confirms that it seems important to take account of the number of items (and of their difficulty) to obtain an accurate formula.

## Competing interests

The authors declare that they have no competing interests.

## Authors' contributions

VS, JBH and BF have made substantial contributions to conception and design, analysis and interpretation of data; FG has been involved in drafting the manuscript and revising it critically for important intellectual content; FB have made substantial contributions to acquisition and interpretation of data; TLN and GK have made substantial contributions to conception and design of the simulation study. All authors read and approved the final manuscript.

## Pre-publication history

The pre-publication history for this paper can be accessed here:

http://www.biomedcentral.com/1471-2288/10/24/prepub

## Supplementary Material

Additional file 1 **Power achieved by the tests of group effects using IRT with fixed person and item difficulty parameters**. Power achieved by the tests of group effects using IRT with fixed person (mean of the latent trait in one group, μ_IRT1_) and item difficulty parameters (good precision: ε = 0.0/moderate precision: ε = 0.5/poor precision: ε = 1.0) as compared with their simulated values for different values of the effect size on the latent trait scale (ES_IRT_), the sample size per group N and the number of items J of the questionnaire. Click here for file

Additional file 2 **Power achieved by the tests of group effects using IRT or CTT**. Power achieved by the tests of group effects using IRT (Rasch model) or CTT in three situations: IRTa (fixed item parameters δ_j _with a good precision (ε = 0.0) and person parameter μ_IRT1 _is estimated)/IRTb (item parameters δ_j _and person parameter μ_IRT1 _are estimated)/CTT for different values of the effect size on the latent trait scale (ES_IRT_), the sample size per group N and the number of items J of the questionnaire. Click here for file

Additional file 3 **Effect size on the score scale (CTT)**. Effect size on the score scale (CTT) for different values of the effect size on the latent trait scale (ES_IRT_), the sample size per group N and the number of items J of the questionnaire. Click here for file

Additional file 4 **Estimated power achieved by the tests of group effects using IRT (Rasch model) or CTT**. Estimated power (1,000 simulations) achieved by the tests of group effects using IRT (Rasch model) or CTT for two different sample sizes per group. Click here for file
