# Supplementary material for: Methodological issues regarding power of classical test theory (CTT) and item response theory (IRT)-based approaches for the comparison of patient-reported outcomes in two groups of patients - a simulation study
Source: BMC Med Res Methodol. 2010 Mar 25;10:24. doi: 10.1186/1471-2288-10-24 (PMC2858729; doi:10.1186/1471-2288-10-24)
Supplement: Additional file 1 — Power achieved by the tests of group effects using IRT with fixed person and item difficulty parameters. Power achieved by the tests of group effects using IRT with fixed person (mean of the latent trait in one group, μIRT1) and item difficulty parameters (good precision: ε = 0.0/moderate precision: ε = 0.5/poor precision: ε = 1.0) as compared with their simulated values for different values of the effect size on the latent trait scale (ESIRT), the sample size per group N and the number of items J of the questionnaire. [file 1471-2288-10-24-S1.DOC]

**Additional file 1.** Power achieved by the tests of group effects using IRT with fixed person (mean of the latent trait in one group, µ_IRT1_) and item difficulty parameters (good precision: ε=0.0 / moderate precision: ε=0.5 / poor precision: ε=1.0) as compared with their simulated values for different values of the effect size on the latent trait scale (ES_IRT_), the sample size per group N and the number of items J of the questionnaire.

|  |  | Number of items J | | | | | |  |
| --- | --- | --- | --- | --- | --- | --- | --- | --- |
| ES_IRT_ | N | 5 | 10 | 15 | 20 | 50 | 100 | Expected Power (IRT)* |
|  |  | Precision: good (ε=0.0) / moderate (ε=0.5) / poor (ε=1.0) | | | | | |  |
| 0.2 | 100 | 0.275/0.261/0.245 | 0.385/0.376/0.361 | 0.409/0.393/0.377 | 0.413/0.408/0.387 | 0.450/0.444/0.432 | 0.511/0.508/0.496 | 0.293 |
|  | 200 | 0.526/0.513/0.485 | 0.612/0.597/0.574 | 0.681/0.667/0.639 | 0.713/0.712/0.682 | 0.739/0.729/0.720 | 0.809/0.802/0.786 | 0.516 |
|  | 300 | 0.694/0.680/0.649 | 0.785/0.774/0.754 | 0.847/0.846/0.819 | 0.868/0.861/0.847 | 0.922/0.914/0.902 | 0.910/0.907/0.897 | 0.688 |
|  | 400 | 0.824/0.814/0.779 | 0.890/0.880/0.874 | 0.930/0.926/0.916 | 0.956/0.948/0.943 | 0.964/0.961/0.959 | 0.970/0.968/0.965 | 0.807 |
|  | 500 | 0.878/0.866/0.843 | 0.957/0.953/0.944 | 0.965/0.963/0.952 | 0.9670.965/0.958 | 0.988/0.987/0.987 | 0.993/0.993/0.990 | 0.885 |
|  | 800 | 0.979/0.974/0.966 | 0.995/0.994/0.993 | 0.999/0.999/0.996 | 0.999/0.999/0.998 | 0.999/0.998/0.998 | 0.999/0.999/0.999 | 0.979 |

IRT: item response theory. *: Power calculations based on the corresponding effect size ES_IRT_ and sample size per group N, with α (two-sided) = 0.05.

**Additional file 1.** cont.

|  |  | Number of items J | | | | | |  |
| --- | --- | --- | --- | --- | --- | --- | --- | --- |
| ES_IRT_ | N | 5 | 10 | 15 | 20 | 50 | 100 | Expected Power (IRT)* |
|  |  | Precision: good (ε=0.0) / moderate (ε=0.5) / poor (ε=1.0) | | | | | |  |
| 0.5 | 100 | 0.937/0.931/0.911 | 0.979/0.981/0.973 | 0.986/0.986/0.981 | 0.994/0.994/0.992 | 0.996/0.996/0.995 | 0.995/0.995/0.994 | 0.942 |
|  | 200 | 0.998/0.996/0.995 | 1.000/1.000/1.000 | 1.000/1.000/1.000 | 1.000/1.000/1.000 | 1.000/1.000/1.000 | 1.000/1.000/1.000 | 0.999 |
|  | 300 | 1.000/1.000/1.000 | 1.000/1.000/1.000 | 1.000/1.000/1.000 | 1.000/1.000/1.000 | 1.000/1.000/1.000 | 1.000/1.000/1.000 | 1.000 |
|  | 400 | 1.000/1.000/1.000 | 1.000/1.000/1.000 | 1.000/1.000/1.000 | 1.000/1.000/1.000 | 1.000/1.000/1.000 | 1.000/1.000/1.000 | 1.000 |
|  | 500 | 1.000/1.000/1.000 | 1.000/1.000/1.000 | 1.000/1.000/1.000 | 1.000/1.000/1.000 | 1.000/1.000/1.000 | 1.000/1.000/1.000 | 1.000 |
|  | 800 | 1.000/1.000/1.000 | 1.000/1.000/1.000 | 1.000/1.000/1.000 | 1.000/1.000/1.000 | 1.000/1.000/1.000 | 1.000/1.000/1.000 | 1.000 |
| 0.8 | 100 | 1.000/1.000/1.000 | 1.000/1.000/1.000 | 1.000/1.000/1.000 | 1.000/1.000/1.000 | 1.000/1.000/1.000 | 1.000/1.000/1.000 | 1.000 |
|  | 200 | 1.000/1.000/1.000 | 1.000/1.000/1.000 | 1.000/1.000/1.000 | 1.000/1.000/1.000 | 1.000/1.000/1.000 | 1.000/1.000/1.000 | 1.000 |
|  | 300 | 1.000/1.000/1.000 | 1.000/1.000/1.000 | 1.000/1.000/1.000 | 1.000/1.000/1.000 | 1.000/1.000/1.000 | 1.000/1.000/1.000 | 1.000 |
|  | 400 | 1.000/1.000/1.000 | 1.000/1.000/1.000 | 1.000/1.000/1.000 | 1.000/1.000/1.000 | 1.000/1.000/1.000 | 1.000/1.000/1.000 | 1.000 |
|  | 500 | 1.000/1.000/1.000 | 1.000/1.000/1.000 | 1.000/1.000/1.000 | 1.000/1.000/1.000 | 1.000/1.000/1.000 | 1.000/1.000/1.000 | 1.000 |
|  | 800 | 1.000/1.000/1.000 | 1.000/1.000/1.000 | 1.000/1.000/1.000 | 1.000/1.000/1.000 | 1.000/1.000/1.000 | 1.000/1.000/1.000 | 1.000 |

IRT: item response theory. *: Power calculations based on the corresponding effect size ES_IRT_ and sample size per group N, with α (two-sided) = 0.05.
