# Supplementary material for: Methodological issues regarding power of classical test theory (CTT) and item response theory (IRT)-based approaches for the comparison of patient-reported outcomes in two groups of patients - a simulation study
Source: BMC Med Res Methodol. 2010 Mar 25;10:24. doi: 10.1186/1471-2288-10-24 (PMC2858729; doi:10.1186/1471-2288-10-24)
Supplement: Additional file 2 — Power achieved by the tests of group effects using IRT or CTT. Power achieved by the tests of group effects using IRT (Rasch model) or CTT in three situations: IRTa (fixed item parameters δj with a good precision (ε = 0.0) and person parameter μIRT1 is estimated)/IRTb (item parameters δj and person parameter μIRT1 are estimated)/CTT for different values of the effect size on the latent trait scale (ESIRT), the sample size per group N and the number of items J of the questionnaire. [file 1471-2288-10-24-S2.DOC]

**Additional file 2.** Power achieved by the tests of group effects using IRT (Rasch model) or CTT in three situations: IRTa (fixed item parameters δ_j_ with a good precision (ε=0.0) and person parameter µ_IRT1_ is estimated) / IRTb (item parameters δ_j_ and person parameter µ_IRT1_ are estimated) / CTT for different values of the effect size on the latent trait scale (ES_IRT_), the sample size per group N and the number of items J of the questionnaire.

|  |  | Number of items J | | | | | |  |
| --- | --- | --- | --- | --- | --- | --- | --- | --- |
| ES_IRT_ | N | 5 | 10 | 15 | 20 | 50 | 100 | Expected Power (IRT)* |
|  |  | Power of the test (IRTa / IRTb / CTT) | | | | | |  |
| 0.2 | 100 | 0.145/0.144/0.144 | 0.222/0.221/0.222 | 0.232/0.231/0.233 | 0.256/0.245/0.255 | 0.268/0.265/0.268 | 0.273/0.269/0.273 | 0.293 |
|  | 200 | 0.297/0.297/0.295 | 0.369/0.368/0.368 | 0.379/0.376/0.380 | 0.433/0.430/0.433 | 0.474/0.465/0.475 | 0.515/0.505/0.514 | 0.516 |
|  | 300 | 0.417/0.418/0.416 | 0.490/0.485/0.489 | 0.568/0.563/0.567 | 0.587/0.585/0.587 | 0.637/0.635/0.638 | 0.663/0.656/0.664 | 0.688 |
|  | 400 | 0.527/0.526/0.526 | 0.602/0.602/0.602 | 0.679/0.682/0.679 | 0.697/0.686/0.697 | 0.777/0.776/0.777 | 0.777/0.777/0.777 | 0.807 |
|  | 500 | 0.597/0.600/0.597 | 0.719/0.718/0.720 | 0.763/0.765/0.763 | 0.804/0.804/0.803 | 0.841/0.834/0.841 | 0.853/0.853/0.853 | 0.885 |
|  | 800 | 0.791/0.792/0.791 | 0.908/0.906/0.908 | 0.933/0.933/0.933 | 0.934/0.934/0.934 | 0.962/0.956/0.961 | 0.973/0.969/0.973 | 0.979 |

IRT: item response theory; CTT: classical test theory; *: Power calculations based on the corresponding effect size ES_IRT_ and sample size per group N, with α (two-sided) = 0.05.

**Additional file 2.** cont.

|  |  | Number of items J | | | | | |  |
| --- | --- | --- | --- | --- | --- | --- | --- | --- |
| ES_IRT_ | N | 5 | 10 | 15 | 20 | 50 | 100 | Expected Power (IRT)* |
|  |  | Power of the test (IRTa / IRTb / CTT) | | | | | |  |
| 0.5 | 100 | 0.699/0.701/0.699 | 0.837/0.835/0.837 | 0.836/0.837/0.835 | 0.877/0.873/0.877 | 0.915/0.912/0.915 | 0.929/0.926/0.928 | 0.942 |
|  | 200 | 0.934/0.932/0.933 | 0.980/0.979/0.980 | 0.989/0.989/0.989 | 0.992/0.993/0.992 | 0.997/0.997/0.997 | 0.996/0.996/0.996 | 0.999 |
|  | 300 | 0.990/0.990/0.990 | 0.999/0.999/0.999 | 0.998/0.999/0.998 | 0.999/0.999/0.999 | 1.000/1.000/1.000 | 1.000/1.000/1.000 | 1.000 |
|  | 400 | 0.998/0.998/0.998 | 1.000/1.000/1.000 | 1.000/1.000/1.000 | 1.000/1.000/1.000 | 1.000/1.000/1.000 | 1.000/1.000/1.000 | 1.000 |
|  | 500 | 1.000/1.000/1.000 | 1.000/1.000/1.000 | 1.000/1.000/1.000 | 1.000/1.000/1.000 | 1.000/1.000/1.000 | 1.000/1.000/1.000 | 1.000 |
|  | 800 | 1.000/1.000/1.000 | 1.000/1.000/1.000 | 1.000/1.000/1.000 | 1.000/1.000/1.000 | 1.000/1.000/1.000 | 1.000/1.000/1.000 | 1.000 |
| 0.8 | 100 | 0.978/0.979/0.978 | 0.994/0.993/0.994 | 0.995/0.995/0.995 | 0.998/0.998/0.998 | 1.000/1.000/1.000 | 0.999/0.999/0.999 | 1.000 |
|  | 200 | 1.000/1.000/1.000 | 1.000/1.000/1.000 | 1.000/1.000/1.000 | 1.000/1.000/1.000 | 1.000/1.000/1.000 | 1.000/1.000/1.000 | 1.000 |
|  | 300 | 1.000/1.000/1.000 | 1.000/1.000/1.000 | 1.000/1.000/1.000 | 1.000/1.000/1.000 | 1.000/1.000/1.000 | 1.000/1.000/1.000 | 1.000 |
|  | 400 | 1.000/1.000/1.000 | 1.000/1.000/1.000 | 1.000/1.000/1.000 | 1.000/1.000/1.000 | 1.000/1.000/1.000 | 1.000/1.000/1.000 | 1.000 |
|  | 500 | 1.000/1.000/1.000 | 1.000/1.000/1.000 | 1.000/1.000/1.000 | 1.000/1.000/1.000 | 1.000/1.000/1.000 | 1.000/1.000/1.000 | 1.000 |
|  | 800 | 1.000/1.000/1.000 | 1.000/1.000/1.000 | 1.000/1.000/1.000 | 1.000/1.000/1.000 | 1.000/1.000/1.000 | 1.000/1.000/1.000 | 1.000 |

IRT: item response theory; CTT: classical test theory; *: Power calculations based on the corresponding effect size ES_IRT_ and sample size per group N, with α (two-sided) = 0.05.
