# Supplementary material for: Methodological issues regarding power of classical test theory (CTT) and item response theory (IRT)-based approaches for the comparison of patient-reported outcomes in two groups of patients - a simulation study
Source: BMC Med Res Methodol. 2010 Mar 25;10:24. doi: 10.1186/1471-2288-10-24 (PMC2858729; doi:10.1186/1471-2288-10-24)
Supplement: Additional file 3 — Effect size on the score scale (CTT). Effect size on the score scale (CTT) for different values of the effect size on the latent trait scale (ESIRT), the sample size per group N and the number of items J of the questionnaire. [file 1471-2288-10-24-S3.DOC]

**Additional file 3.** Effect size on the score scale (CTT) for different values of the effect size on the latent trait scale (ES_IRT_), the sample size per group N and the number of items J of the questionnaire.

|  |  | Number of items J | | | | | |
| --- | --- | --- | --- | --- | --- | --- | --- |
| ES_IRT_ | N | 5 | 10 | 15 | 20 | 50 | 100 |
| 0.2 | 100 | 0.134 (0.136) | 0.165 (0.145) | 0.170 (0.138) | 0.178 (0.140) | 0.188 (0.139) | 0.197 (0.138) |
|  | 200 | 0.141 (0.098) | 0.161 (0.100) | 0.167 (0.098) | 0.177 (0.102) | 0.183 (0.103) | 0.194 (0.102) |
|  | 300 | 0.141 (0.082) | 0.160 (0.080) | 0.175 (0.079) | 0.178 (0.081) | 0.190 (0.083) | 0.193 (0.082) |
|  | 400 | 0.143 (0.070) | 0.156 (0.072) | 0.173 (0.071) | 0.175 (0.071) | 0.188 (0.069) | 0.189 (0.069) |
|  | 500 | 0.140 (0.063) | 0.160 (0.061) | 0.170 (0.064) | 0.175 (0.063) | 0.190 (0.065) | 0.193 (0.063) |
|  | 800 | 0.138 (0.048) | 0.160 (0.048) | 0.172 (0.050) | 0.178 (0.051) | 0.186 (0.051) | 0.186 (0.051) |
| 0.5 | 100 | 0.345 (0.140) | 0.402 (0.135) | 0.415 (0.140) | 0.432 (0.134) | 0.467 (0.132) | 0.473 (0.133) |
|  | 200 | 0.348 (0.100) | 0.398 (0.095) | 0.418 (0.099) | 0.432 (0.096) | 0.464 (0.093) | 0.474 (0.098) |
|  | 300 | 0.346 (0.081) | 0.398 (0.078) | 0.429 (0.080) | 0.435 (0.077) | 0.463 (0.077) | 0.469 (0.074) |
|  | 400 | 0.348 (0.069) | 0.400 (0.070) | 0.417 (0.065) | 0.434 (0.067) | 0.462 (0.066) | 0.475 (0.068) |
|  | 500 | 0.342 (0.061) | 0.400 (0.060) | 0.421 (0.058) | 0.432 (0.060) | 0.462 (0.060) | 0.474 (0.061) |
|  | 800 | 0.343 (0.050) | 0.396 (0.049) | 0.421 (0.047) | 0.431 (0.047) | 0.465 (0.047) | 0.473 (0.048) |

Data are average (SD) of the effect size on the score scale; CTT: classical test theory

**Additional file 3.** cont.

|  |  | Number of items J | | | | | |
| --- | --- | --- | --- | --- | --- | --- | --- |
| ES_IRT_ | N | 5 | 10 | 15 | 20 | 50 | 100 |
| 0.8 | 100 | 0.548 (0.135) | 0.621 (0.127) | 0.649 (0.126) | 0.673 (0.126) | 0.706 (0.124) | 0.724 (0.120) |
|  | 200 | 0.548 (0.094) | 0.622 (0.091) | 0.655 (0.090) | 0.670 (0.088) | 0.714 (0.085) | 0.734 (0.090) |
|  | 300 | 0.539 (0.075) | 0.618 (0.073) | 0.653 (0.071) | 0.673 (0.072) | 0.712 (0.072) | 0.727 (0.070) |
|  | 400 | 0.540 (0.063) | 0.619 (0.063) | 0.648 (0.064) | 0.675 (0.061) | 0.715 (0.061) | 0.729 (0.064) |
|  | 500 | 0.541 (0.062) | 0.615 (0.057) | 0.651 (0.056) | 0.672 (0.054) | 0.714 (0.055) | 0.728 (0.055) |
|  | 800 | 0.543 (0.046) | 0.618 (0.046) | 0.654 (0.046) | 0.675 (0.043) | 0.715 (0.043) | 0.731 (0.041) |

Data are average (SD) of the effect size on the score scale; CTT: classical test theory
