# Supplementary material for: Methodological issues regarding power of classical test theory (CTT) and item response theory (IRT)-based approaches for the comparison of patient-reported outcomes in two groups of patients - a simulation study
Source: BMC Med Res Methodol. 2010 Mar 25;10:24. doi: 10.1186/1471-2288-10-24 (PMC2858729; doi:10.1186/1471-2288-10-24)
Supplement: Additional file 4 — Estimated power achieved by the tests of group effects using IRT (Rasch model) or CTT. Estimated power (1,000 simulations) achieved by the tests of group effects using IRT (Rasch model) or CTT for two different sample sizes per group. [file 1471-2288-10-24-S4.DOC]

**Additional file 4.** Estimated power (1,000 simulations) achieved by the tests of group effects using IRT (Rasch model) or CTT for two different sample sizes per group.

| Number of patients /group | Method of analyse | Estimated power |
| --- | --- | --- |
| 195 | IRT | 0.789 |
| 195 | CTT | 0.782 |
| 287 | IRT | 0.913 |
| 287 | CTT | 0.906 |

IRT: item response theory; CTT: classical test theory
